# Comparison of inhibitory effects and mechanisms of lactonic sophorolipid on different pathogenic bacteria

**DOI:** 10.3389/fmicb.2022.929932

**Published:** 2022-09-27

**Authors:** Xiao-jing Ma, Tong Wang, Hui-min Zhang, Jun-qian Shao, Mei Jiang, Huai Wang, Hui-xia Zhu, Dong Zhou

**Affiliations:** ^1^School of Food and Biological Engineering, Hefei University of Technology, Hefei, China; ^2^Ministry of Education, Engineering Research Center of Bio-Process, Hefei University of Technology, Hefei, China; ^3^Department of Pediatrics, Qilu Hospital of Shandong University, Jinan, China

**Keywords:** lactonic sophorolipid, pathogenic bacteria, antibacterial effect, antibacterial mechanism, *Staphylococcus aureus*, *Pseudomonas aeruginosa*

## Abstract

Crude sophorolipids (SLs) have been proven to perform varying degrees of inhibitory effects on different pathogenic bacteria. However, systematic comparative studies of pure lactonic sophorolipid (LSL) among different types of bacteria are few. In this study, the antibacterial effects and mechanisms of LSL on pathogenic bacteria of *Staphylococcus aureus*, *Lactobacillus* sp., *Pseudomonas aeruginosa*, and *Escherichia coli* were investigated. Bacteriostatic circle, antibacterial rate, minimum inhibitory concentration (MIC), and minimum bactericidal concentration (MBC) of LSL on different pathogenic bacteria were measured. Then, the antibacterial mechanisms of LSL on *S. aureus* and *P. aeruginosa* were explored using ultrastructural observation, cell membrane permeability analysis, intracellular ATP content determination, and extracellular UV absorption detection. With the minimum MIC and MBC values of 0.05 and 0.20 mg/ml, LSL exhibited the best inhibitory effect against *S. aureus*, followed by *P. aeruginosa*. LSL showed no significant inhibitory effect on *E. coli* and *Lactobacillus* sp. For both *S. aureus* and *P. aeruginosa*, LSL achieved bacteriostatic and bactericidal effects by destroying the cell wall, increasing the permeability of the cell membrane and leading to the flow out of intracellular contents. However, the action mode and action intensity of LSL on the cell wall and membrane of these two bacteria were significantly different. LSL had a greater influence on the cell membrane of *S. aureus* by “leaking,” while it exhibited a stronger effect on the cell wall of *P. aeruginosa* by “blasting.” These results contributed to a better understanding of the relationship between LSL and different bacterial cell structures, further suggesting the conclusion that LSL might be used for the targeted treatment of special pathogenic bacteria.

## Introduction

Sophorolipids (SLs), mainly produced by different species of *Candida*, are considered the most promising type of biosurfactant. Nowadays, SLs have attracted global attention due to their good surface activity, excellent bactericidal and antifungal properties, biocompatibility, and low toxicity (Huang et al., 2020; Banat et al., 2021). Generally, SL molecules are divided into lactonic sophorolipid (LSL) and acidic sophorolipid (ASL), which have significantly different physicochemical properties and biological activities (Ma et al., 2012). Structurally, all of the SL molecules are composed of a hydrophilic disaccharide head and a hydrophobic fatty acid tail. The differences among them lie in the length of the fatty acid chain, the number of unsaturated bonds, and the degree of acetylation on the sophorose molecule (Van Bogaert et al., 2011). Functionally, LSL performs better antibacterial/bactericidal, antitumor, antiviral, and other pharmacological activities due to the lactone ring structure and can be applied as a biologically active substance in the medical field. ASL shows a lower critical micelle concentration (CMC) value and higher water solubility due to the open-loop structure and mainly be used as pharmaceutical excipients in medical field (Hirata et al., 2009; Shao et al., 2012).

At present, many reports have confirmed the antibacterial properties of crude SLs. As reported by Hu et al. (2012), SLs obtained from oleic acid and glucose could inhibit the growth of pathogenic microorganisms such as *Staphylococcus aureus*, *Escherichia coli*, *Bacillus subtilis*, and *Pseudomonas putida*. With a minimum inhibitory concentration (MIC) of 1.56 mg/ml, SLs exhibited the most remarkable inhibitory effect on *S. aureus*. It was also reported that SLs obtained from soybean oil refinery residue and glutamic acid could inhibit the growth of pathogenic microorganisms such as *Pseudomonas aeruginosa*, *Staphylococcus aureus*, *Candida albicans*, and *Escherichia coli*. When the concentration of SLs was 0.012 mg/ml, the growth inhibition rates of SLs against *S. aureus* and *E. coli* were 15 and 5%, respectively (Rufino et al., 2011). Shao’s (2010) findings revealed that SLs produced from rapeseed oil and glucose had inhibitory effects on *Staphylococcus aureus*, *Bacillus cereus*, and *Streptococcus mutans*. However, SLs showed the best inhibitory effect on *S. aureus* with a minimum MIC value of 0.04 mg/ml and no inhibitory effect on *E. coli*. Besides, Kim et al. (2002) also investigated the inhibitory effect of SLs produced from rapeseed oil and glucose on *Bacillus subtilis*, *Staphylococcus xylose*, *Streptococcus mutans*, and *Propionibacterium acnes*. With the lowest MIC value of 0.04 mg/ml, SLs had the best inhibition effect on *B. subtilis*, but they had no inhibition effect on *E. coli.* Furthermore, Dengle-Pulate et al. (2014) reported that SLs fermented from lauryl alcohol and glucose had MIC values of 0.03 and 0.01 mg/ml against *E. coli* and *P. aeruginosa*, respectively.

In terms of the antibacterial mechanism of SLs, Kim et al. (2002) confirmed the distinguishing inhibitory effects of SLs on different bacteria and speculated that the difference in antibacterial activity was due to differences in cell wall structures. Hu et al. (2012) mentioned that SLs performed bacteria-killing effects against *S. aureus* mainly by destroying the structure of bacterial cell membrane and cell wall. Besides, it had also been suggested that SLs played antibacterial effects by disrupting biofilms and preventing the formation of biofilms of *S. aureus* and *P. aeruginosa* (Díaz De Rienzo et al., 2016; Pontes et al., 2016; Nguyen et al., 2020).

From the above investigation, we can find that there are still several problems, such as the source of crude SLs is not uniform, the inhibitory effect on the same bacteria is inconsistent, and the antibacterial mechanism is less studied. Therefore, the antibacterial effect and mechanism of SLs on various bacteria need to be further systematically compared and analyzed. In this study, pure LSL was obtained and prepared by our laboratory first, and then the inhibitory effects of LSL against *S. aureus*, *Lactobacillus* sp., *P. aeruginosa*, and *E. coli* were investigated. Finally, the antibacterial mechanism of LSL on *S. aureus* and *P. aeruginosa* were explored and elaborated.

## Materials and methods

### Strains and cultivation

*Staphylococcus aureus* ATCC25923, *Pseudomonas aeruginosa* ATCC9027, *Escherichia coli* ATCC25922, and *Lactobacillus* sp. ATCC7469 were purchased from American Type Culture Collection (ATCC) and preserved at -70°C in our laboratory. Before use, these strains were activated in LB liquid medium to exponential phase at 37°C and 220 rpm.

### Preparation of lactonic sophorolipid and lactonic sophorolipid stock solution

Crude SLs were produced by fermentation of *Starmerella bombicola* CGMCC 1576 with glucose and oleic acid as carbon sources. LSL, with a purity of 95%, was obtained by further separation and purification according to the method reported earlier (Ma et al., 2012). First, the stock solution of LSL with a concentration of 120 g/L was prepared by dissolving LSL in a minimum amount of ethanol. Then, the LSL stock solution was filtered with a 0.22 μm filter membrane for sterilization. In the following antibacterial experiments, a suitable volume of LSL stock solution was taken and diluted with different volumes of autoclaved water/medium to the required LSL concentration.

### Measurement of inhibition zone diameter

At an inoculum dose of 2% (v/v), four pathogenic bacteria were inoculated into LB liquid media, respectively. Inhibition zone diameter was measured using the Oxford-cup method with minor modifications (Chen et al., 2020). Briefly, 100 μl of bacterial culture was spread evenly on the surface of the LB solid medium, then Oxford cups were placed and 200 μl of distilled water or LSL solution at different concentrations was transferred to the cups, respectively. After that, all the bacteria plates were placed in an incubator and cultivated at 37°C for a certain time. Finally, the appearance and diameter of the inhibition zone of each plate were observed and recorded.

### Determination of antibacterial efficiency rate

First, different volumes of LSL stock solution were pipetted into 50 ml of autoclaved LB liquid medium in a 300 ml Erlenmeyer flask and mixed well. Then, the seed liquid of different pathogens with the same concentration of 10^7^ CFU/ml was incubated in the above flask at an inoculum dose of 2% (v/v) and cultivated for 24 h at 37°C. The medium without LSL addition was used as a blank control. Samples were taken at different times, and the OD values were determined at 600 nm (Nguyen et al., 2020). The antibacterial efficiency rate (AER) of different concentrations of LSL at different times was calculated using the following equation:


Antibacterialefficiencyrate(AER,%)

$$=\left({\text{OD}}_{\text{blank}}-{\text{OD}}_{\text{LSL}}\right)/{\text{OD}}_{\text{blank}}\times 100$$

### Determination of minimum inhibitory concentration and minimum bactericidal concentration

The MIC and MBC values of LSL against *S. aureus* and *P. aeruginosa* were determined in 6-well plates. First, 2 ml of LB liquid medium containing the required LSL concentration was added to the well. Then, 200 μl of *S. aureus* and *P. aeruginosa* culture in the concentration of 10^7^CFU/ml were inoculated, respectively. After cultivation at 37°C for 24 h, the OD_600_ of the bacterial solutions was measured and recorded. Subsequently, 100 μl of the above bacterial culture was taken and evenly spread on a fresh LB solid medium, respectively. MIC of LSL against *S. aureus* or *P. aeruginosa* was defined as the specified concentration when the change in OD value was less than 5% compared with the initial OD value. MBC of LSL against *S. aureus* or *P. aeruginosa* was defined as the lowest concentration at which no colony growth was observed on the LB solid medium (Garba and Hafsat, 2013).

### Scanning electron microscopy (SEM) observation

Observation of the morphological changes in the *S. aureus* and *P. aeruginosa* cells was performed using SEM (Joshi-Navare and Prabhune, 2013). Bacteria in the exponential growth phase were collected and treated with the LSL at the MBC level or untreated as the control. These suspensions were placed at 37°C for a total incubation time of 24 h, and different samples were taken at different times (0, 4, 6, 8, and 24 h). The bacterial pellets were harvested by centrifugation at 4°C. After washing with 0.1 M PBS buffer (pH 7.2) 3 times and fixing with 2.5% glutaraldehyde at 4°C for 12 h, the cell pellets were dehydrated with gradients of 50, 70, 80, 90, and 100% ethanol. Then, the dehydrated samples were dried in a vacuum freeze dryer (HX-10N-50B, Shanghai Hushi Industrial Co., Ltd., China) for 6 h and coated with gold by a Baltec SCD050 Sputter coater. The micrographs were obtained using a TESCAN MIRA3 scanning electron microscope (Tescan, Czech).

### Cell membrane permeability analysis

The fluorescent probes of carboxyfluorescein diacetate [5(6)-Cfda] and propidium iodide (PI) (Rockville, United States) were used to distinguish live cells from dead cells (Hoefel et al., 2003). First, *S. aureus* and *P. aeruginosa* in the logarithmic growth phase were centrifuged at 4°C to collect the bacterial pellet. After washing with a 0.75% NaCl solution, the cell pellet was divided into two parts. Half were given LSL solution at the MBC level and the other half were treated with an equal volume of saline as the control. After cultivation for an additional 24 h, the bacteria pellet was harvested and resuspended in 500 μl of saline, then cFDA and PI with final concentrations of 100 and 30 μmol/L were added in sequence and reacted for 10 min. These cell pellets were harvested by centrifugation and resuspended in 500 μl of saline again. Finally, 3.0 μl of the bacterial suspension was taken and placed under the FV1000 confocal laser scanning microscope (Olympus, Japan) for observation.

### Intracellular ATP content determination

Bacterial suspensions of *S. aureus* and *P. aeruginosa* were prepared in the same method as described above for the SEM observation. During the cultivation period, two samples were taken at each time point. One was centrifuged to collect bacterial pellet for intracellular ATP content measurement according to the instructions of the ATP assay kit (Solarbio, China), and the other was centrifuged and the supernatant was retained for the following determination of extracellular ultraviolet absorption substances.

### Extracellular ultraviolet absorption substance measurement

The supernatant samples of LSL-treated *S. aureus* and *P. aeruginosa* prepared above were used for drawing the variation curve of extracellular ultraviolet absorbing substances (Shi et al., 2016). The concentration of extracellular ultraviolet substances was determined at 260 nm by an ultraviolet spectrophotometer (UV-VIS, Hitachi High-Tech, Japan). The degree of leakage was expressed by the changes in OD_260_ values at different time points.

### Statistical analysis

All experiments were carried out in triplicate, and the results were expressed as mean ± SD. The data obtained were subjected to a one-way analysis of variance (ANOVA) to determine differences among strains and LSL treatment or not. The statistical program GraphPad Prism 8.0 was employed for statistical analysis and graph drawing.

## Results

### Comparison of the size of inhibition zone of lactonic sophorolipid against different pathogenic bacteria

The Oxford cylinder method was used to compare the antibacterial effects of LSL against gram-positive bacteria of *S. aureus* and *Lactobacillus* sp., and gram-negative bacteria of *P. aeruginosa* and *E. coli*. The size of the inhibition zone produced by LSL against different pathogenic bacteria was significantly different (Table 1). Among them, LSL had the best antibacterial effect on *S. aureus*, followed by *P. aeruginosa*. The diameter of the inhibition zone at 11.25 mm against *S. aureus* was obtained from the plate with 0.50 mg/ml of LSL addition. When LSL concentration increased to 3.13 mg/ml, the inhibition zone began to appear on the *P. aeruginosa* plate, and the diameter was approximately 7.30 mm. For *Lactobacillus* sp. and *E. coli*, there was still no inhibition zone appearing on the corresponding plate even when the concentration of LSL was increased to 25.00 mg/ml. Contrary to a previous report by Shah and Prabhune (2007), these results showed that LSL had no consistent antibacterial effects against gram-positive bacteria or gram-negative bacteria. In addition, since LSL was ineffective against *Lactobacillus* sp. and *E. coli*, we only investigated and compared the bacteriostatic effects of LSL against *S. aureus* and *P. aeruginosa* in the subsequent bacteriostatic experiments.

**TABLE 1:** Inhibitory effects of LSL on four pathogens using the Oxford cup method.

*S. aureus*	LSL (mg/ml)	0	0.50	1.00	2.00	5.00
	
	Inhibition zone diameter (mm)	0	11.25 ± 0.12	12.50 ± 0.10	13.35 ± 0.15	15.05 ± 0.05

*Lactobacillus* sp.	LSL (mg/ml)	0	3.13	6.25	12.50	25.00
	
	Inhibition zone diameter (mm)	0	0	0	0	0

*P. aeruginosa*	LSL (mg/ml)	0	0.78	1.56	3.13	6.25
	
	Inhibition zone diameter (mm)	0	0	0	7.30 ± 0.05	12.40 ± 0.07

*E. coli*	LSL (mg/ml)	0	3.13	6.25	12.50	25.00
	
	Inhibition zone diameter (mm)	0	0	0	0	0

### Comparison of the antibacterial rate of lactonic sophorolipid against *Staphylococcus aureus* and *Pseudomonas aeruginosa*

**FIGURE 1:**
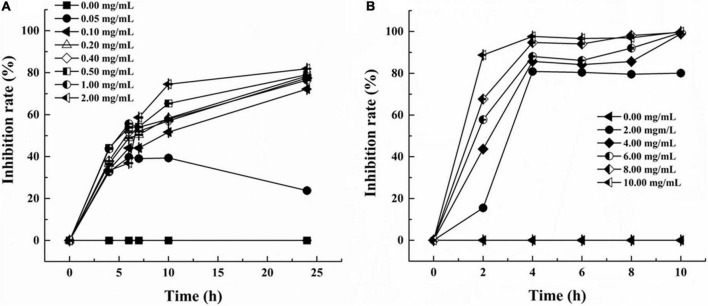
Inhibition rate of LSL at different concentrations against *S. aureus* (A) and *P. aeruginosa* (B).

The inhibition curves of *S. aureus* and *P. aeruginosa* at various concentrations of LSL are shown in Figure 1. Inhibition rates of higher than 30% could be achieved at any concentration of LSL used. LSL concentration required for inhibition of *S. aureus* was much smaller than that of *P. aeruginosa*. We also found that 0.05 mg/ml of LSL could perform a significant inhibitory effect against *S. aureus*. For *P. aeruginosa*, the initial concentration of 2.00 mg/ml of LSL was required to show the inhibitory effect.

Besides, when comparing the inhibition effects of LSL at the same treating time, we found that LSL was more effective against *P. aeruginosa* than *S. aureus*. Taking the time point of 4 h as an example, the inhibition rate of LSL at 2.00 mg/ml on *S. aureus* and *P. aeruginosa* was 33.39 and 80.86%, respectively. The antibacterial rate of LSL against *P. aeruginosa* increased rapidly in the first 2 h and almost reached the maximum at 4 h for all the LSL concentrations from 4.00 to 10.00 mg/ml. The inhibition curves of LSL against *S. aureus* were dose- and time-dependent. As time and concentration increased, the antibacterial rate also increased. At the time point of 24 h, LSL at the concentration of 2.00 mg/ml provided the highest antibacterial rate of 82.31% against *S. aureus*. However, all the LSL concentrations from 0.20 to 2.00 mg/ml exhibited a similar antibacterial rate of approximately 80% against *S. aureus*. The antibacterial rates could not reach 100%, even if the treatment concentration and time continued to increase. This phenomenon was partly attributed to the fact that the dead *S. aureus* cells deposited in the lower layer affected the absorbance of the mixture. Other detailed reasons were discussed in the following sections.

### Comparison of minimum inhibitory concentration and minimum bactericidal concentration of lactonic sophorolipid on *Staphylococcus aureus* against *Pseudomonas aeruginosa*

**FIGURE 2:**
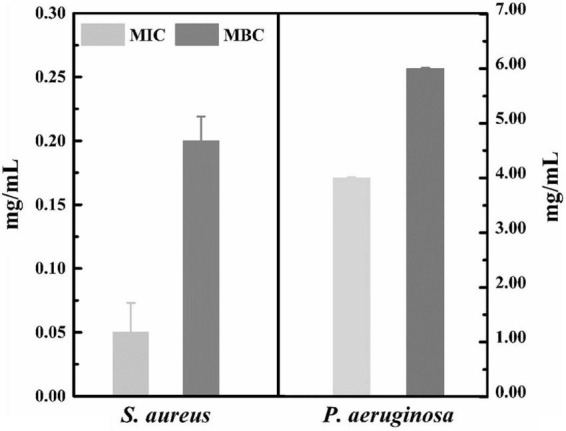
MIC and MBC of LSL against *S. aureus* and *P. aeruginosa.*

The MIC and MBC values of LSL against *S. aureus* and *P. aeruginosa* are shown in Figure 2. LSL performed a better inhibition effect against *S. aureus* than *P. aeruginosa*. The MIC and MBC values of LSL on *S. aureus* were 0.05 and 0.20 mg/ml, which were 80 times and 30 times lower than that of *P. aeruginosa*, respectively. This might be related to the fact that LSL had different effects on these two bacteria in the planktonic microbe state or biofilm state. The antibacterial effects of LSL on *S. aureus* in planktonic microbe state were stronger than that of in biofilm state, while the opposite was for *P. aeruginosa* (Diaz De Rienzo et al., 2016).

### Effects of lactonic sophorolipid on ultrastructure observation of *Staphylococcus aureus* and *Pseudomonas aeruginosa*

**FIGURE 3:**
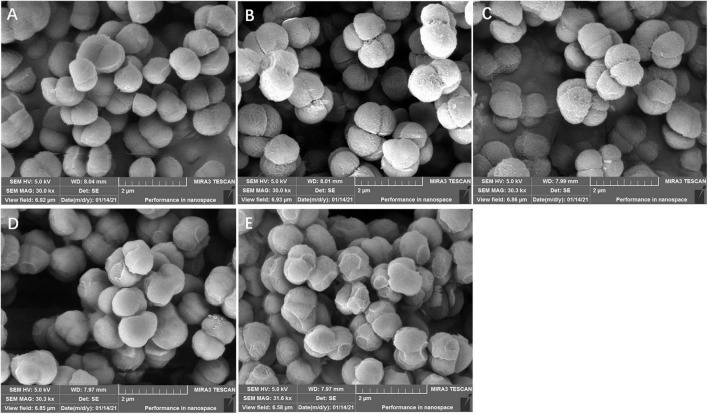
Structural morphology changes observation of *S. aureus* before and after LSL treatment at different time intervals. SEM images of untreated *S. aureus* cells (A) and LSL treated for 4 h (B), 6 h (C), 8 h (D), and 24 h (E) cells.

**FIGURE 4:**
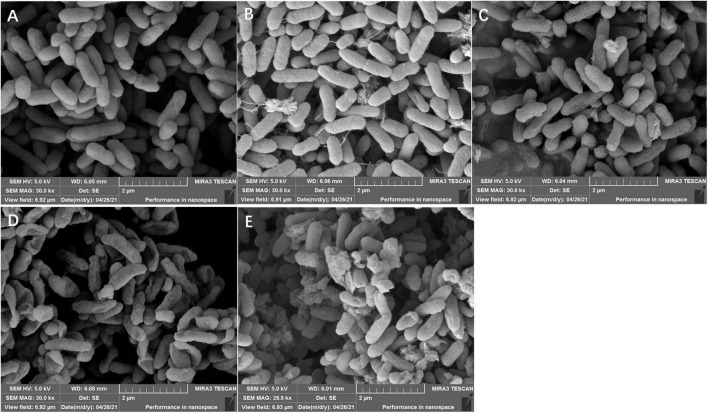
Structural morphology changes observation of *P. aeruginosa* before and after LSL treatment at different time intervals. SEM images of untreated *P. aeruginosa* cells (A) and LSL treated for 4 h (B), 6 h (C), 8 h (D), and 24 h (E) cells.

As shown in Figures 3, 4, LSL treatment caused varying degrees and manners of the destruction of cell walls of *S. aureus* and *P. aeruginosa*. The untreated cells of *S. aureus* and *P. aeruginosa* appeared fully globose or rod-shaped without obvious abnormalities, and there were distinct boundaries between the cells (Figures 3A, 4A). After treatment with 0.20 mg/ml of LSL for 4–8 h, the surface of *S. aureus* cells became rougher, the body began to shrink, and a small amount of cell deformation could be observed at the later stage of LSL treatment (Figures 3B–D). When treated with LSL for 24 h, *S. aureus* cells shrank more severely, and most cells were unable to maintain their original shape and integrity due to their serious “leaking” and shrinkage (Figure 3E).

For *P. aeruginosa* cells treated with 6.00 mg/ml of LSL after 4–8 h, more “blasting” bacterial cells appeared. The number of bacteria with “blasting” holes increased with the extension of treatment time, eventually failing to maintain the cell integrity (Figures 4B–D). After being treated with LSL for 24 h, *P. aeruginosa* cells were unable to maintain the complete rod-like structure due to the “blasting” holes, and the severely damaged bacteria appeared to agglomerate (Figure 4E). It was speculated that LSL increased the permeability of the cell wall and cell membrane of *S. aureus*, leading to an outflow of cellular contents, subsequent cell shrinkage, and eventual cell death. While LSL might affect and hinder the synthesis of the cell wall of *P. aeruginosa*, this might inhibit the cell growth by preventing it from successfully synthesizing new intact cell walls (Nguyen et al., 2020).

### Cell membrane permeability analysis of lactonic sophorolipid-treated *Staphylococcus aureus* and *Pseudomonas aeruginosa*

**FIGURE 5:**
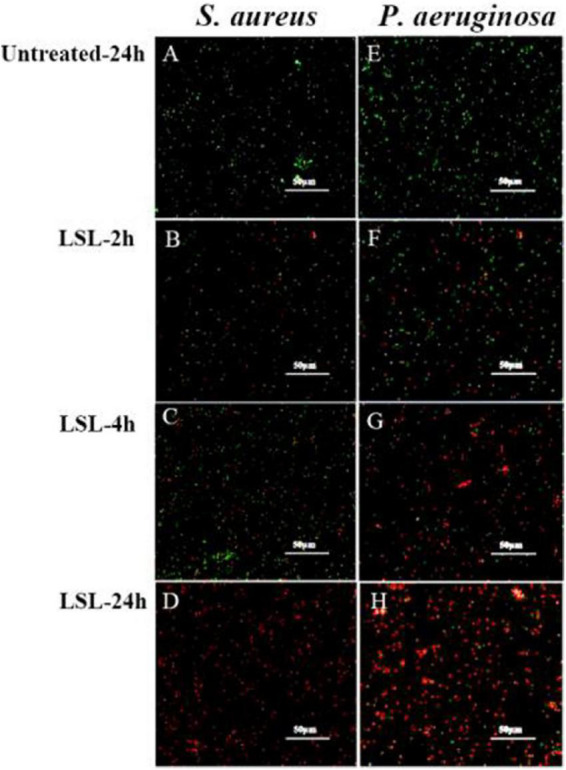
Comparison of fluorescence information changes in *S. aureus* and *P. aeruginosa* by CLSM after LSL treatment at different time intervals. Figures 5A–D showed CLSM images of *S. aureus* cells without treatment (A) and treated with LSL for 2 h (B), 4 h (C), and 24 h (D). Figures 5E–H showed CLSM images of P. aeruginosa cells without treatment (E) and treated with LSL for 2 h (F), 4 h (G), and 24 h (H).

Combined with the use of CLSM, fluorescent probes of cFDA and PI were used for distinguishing the live cells from dead cells. cFDA could enter living cells and emit green fluorescence, while PI could combine with damaged cells to emit red fluorescence (Hoefel et al., 2003). In this study, both untreated *S. aureus* and untreated *P. aeruginosa* grew well and showed no red fluorescence (Figures 5A,E). More and more damaged cells exhibiting red fluorescence were observed with the extension of the LSL treating time. Furthermore, it was found that the intensity of red fluorescence in S. aureus within 2–4 h (Figures 5B,C) was significantly weaker than that in *P. aeruginosa* within 2–4 h (Figures 5F,G), indicating that the inhibitory effect of LSL on *P. aeruginosa* was better than that of *S. aureus* in a short period of time. However, at the time point of 24 h, there was almost no green fluorescence in *S. aureus* cells (Figure 5D), while there was still some green fluorescence in *P. aeruginosa* cells (Figure 5H), suggesting that LSL had a better long-term antibacterial effect on *S. aureus*. These cell membrane permeability results were consistent with the inhibition effect of LSL on the growth of *S. aureus* and *P. aeruginosa* in Figure 1.

### Changes in intracellular ATP content of lactonic sophorolipid-treated *Staphylococcus aureus* and *Pseudomonas aeruginos*

**FIGURE 6:**
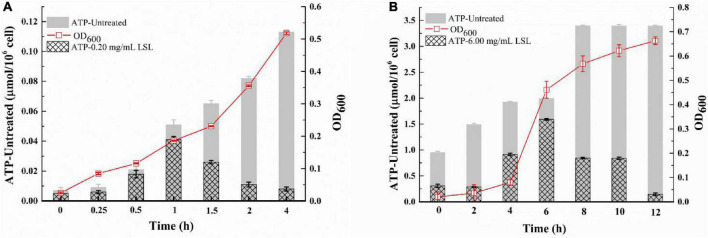
Changes in intracellular ATP content of *S. aureus* (A) and *P. aeruginosa* (B) before and after LSL treatment.

The integrity of the bacterial membrane can be inferred by measuring the changes in ATP content in bacterial cells (Shi et al., 2016). As shown in Figure 6A, the intracellular ATP content of the untreated *S. aureus* cells continued to increase as time went on, and the intracellular ATP content of LSL-treated *S. aureus* was always lower than that of the untreated group. The trend of intracellular ATP content of *S. aureus* was increasing and then decreasing with the prolongation of treating time. This phenomenon was related to the growth trend of *S. aureus*. LSL merely showed a relatively weak effect on *S. aureus* cells during the early stage of LSL treatment (0–1 h). The reason lies in the fact that the destructive and leaking effects of LSL were weaker than the growth ability of *S. aureus*. Hence, the total intracellular ATP content was still rising. With the prolongation of LSL treatment time (1–4 h), the destructive and leaking effect of LSL on *S. aureus* gradually exceeded the growth rate of the bacteria, so the intracellular ATP content exhibited a downward trend. The changing trend of the intracellular ATP content of LSL-treated *P. aeruginosa* was similar to that of *S. aureus*, except that *P. aeruginosa* took a much longer time of 12 h (Figure 6B).

These results indicated that LSL could disrupt the integrity of cell membranes of both *S. aureus* and *P. aeruginosa*, leading to leakage of intracellular ATP. However, the loss of ATP in *S. aureus* was faster, and the time required for all the ATP outflow was shorter when compared with *P. aeruginosa*. These results suggest that LSL had a stronger damaging effect on the cell membrane of *S. aureus* than *P. aeruginosa*. Again, these results helped us further explain why the morphological changes in *S. aureus* and *P. aeruginosa* cells were so distinct (Figures 3, 4).

### Detection of extracellular ultraviolet absorbing substances of lactonic sophorolipid-treated *Staphylococcus aureus* and *Pseudomonas aeruginosa*

**FIGURE 7:**
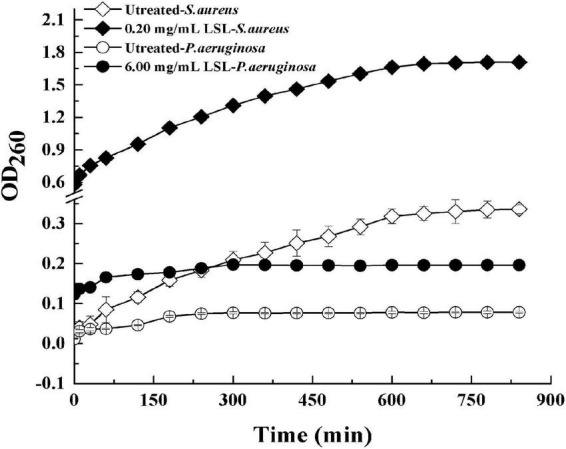
Changes in extracellular ultraviolet absorption substances of *S. aureus* and *P. aeruginosa* before and after LSL treatment.

Extracellular ultraviolet-absorbing substances mainly include protein, nucleic acid, and other macromolecular substances. In general, these substances cannot escape unless the cell membrane is damaged. The macromolecules would flow out and increase OD_260_ once the cell membrane was damaged (Alakomi et al., 2000). Effects of LSL treatment on the extracellular UV-absorbing substances of *S. aureus* and *P. aeruginosa* are shown in Figure 7.

It was found that the extracellular OD_260_ values of both *S. aureus* and *P. aeruginosa* treated with LSL were significantly higher than those of untreated groups. That was, LSL caused the outflow of intracellular nucleic acid, protein, and other substances by destroying the cell membranes of *S. aureus* and *P. aeruginosa*. Furthermore, we also found that OD_260_ values of LSL-treated *S. aureus* were markedly higher than that of LSL-treated *P. aeruginosa* at any given time point, suggesting LSL performed more destructively on the cell membrane of *S. aureus* than *P. aeruginos*. Again, these results were mutually verified with the results mentioned in intracellular ATP content detection.

## Discussion

Nowadays, biosurfactants of SLs have attracted increasing attention worldwide. More theoretical and applied research related to SLs had been carried out for their excellent surface activity as well as effective antibacterial and antitumor activities (Ma et al., 2012). Many reports have confirmed the inhibitory effects of SLs on various pathogenic bacteria (Solaiman et al., 2017). However, some questions, such as SLs from the same carbon substrates performed different inhibitory effects on the same bacteria in different reports, and the antibacterial mechanism had not been clearly described, are still needed to be resolved. These phenomena were partly caused by the complex composition of the crude SLs obtained from inconsistent fermentation methods (Chen et al., 2014), the difficulty in separation and purification of crude SLs (Rosenberg and Ron, 1999), and the trouble with standard product purchasing. To further clarify the inhibitory effect and mechanism of SLs on different pathogenic bacteria, LSL with purity over 95% was prepared and used in this study.

When comparing the inhibitory effects of LSL on gram-positive bacteria of *S. aureus* and *E. coli*, as well as gram-negative bacteria of *P. aeruginosa* and *Lactobacillus* sp., we found that LSL showed the best inhibitory effect on *S. aureus*, then on *P. aeruginosa*, and basically no inhibitory effect on *E. coli* and *Lactobacillus* sp. These results were consistent with the results of Shah et al. (2007), while obviously different from the study of Dengle-Pulate et al. (2014); they reported that SLs could inhibit the growth of *E. coli* at a low concentration of 0.03 mg/ml. Besides, Shah and Prabhune (2007) considered that the inhibitory effects of SLs on gram-positive bacteria were greater than on gram-negative bacteria. However, our present results showed that LSL was valid only for some specific bacteria, but not for the bacteria being gram-positive or gram-negative.

When further exploring the antibacterial mechanism of LSL on different bacteria, we found that the action mode and action intensity of LSL on the cell wall and membrane of *S. aureus* and *P. aeruginosa* were quite different. By SEM observations, we found *S. aureus* mainly appeared in “leaking and shrinkage,” while *P. aeruginosa* mainly appeared in “blasting.” LSL played the antibacterial role by increasing the permeability of the cell wall of *S. aureus*, causing the flow out of cell contents, resulting in the “shrinkage” of cells and eventually the inhibition of growth. For *P. aeruginosa*, LSL acted by hindering the synthesis of the cell wall, causing the failure to synthesize new and complete cell walls, resulting in the “exploding” of cells and eventually death (Nguyen et al., 2020). In other words, LSL performed a stronger destructive effect on the cell wall of *P. aeruginosa* than that of *S. aureus*.

These different behaviors were the consequence of the different structures of the cell wall between *S. aureus* and *P. aeruginosa*. The cell wall of *S. aureus* is thick and mainly composed of peptidoglycan and teichoic acid. As a biosurfactant with a macrolide structure, it was difficult for LSL to interact with the cell wall of *S. aureus*. In addition, LSL might mainly promote the formation of biosurfactant-enriched domains within the phospholipid bilayer and inhibit the protein synthesis function of the cell membrane (Kaneko et al., 2007; An et al., 2020; Marcelino et al., 2021). But for *P. aeruginosa*, the peptidoglycan layer of the cell wall is thin, the cross-linking is loose, and the lipid content is high (Rajivgandhi et al., 2018). Higher lipid content within the cell wall of *P. aeruginosa* made LSL easier to fuse with, thus preventing the formation of a new cell wall and destroying the cell membrane structure (Breiner-Goldstein et al., 2021).

Similar to the results reported by Sana et al. (2018), we also found that the cell membrane was another action site of LSL by cell membrane permeability analysis experiments of intracellular ATP content measurement and extracellular UV absorption substance detection. It was reported that SL-induced membrane permeabilization and content leakage could be the result of the formation of laterally segregated domains (Marcelino et al., 2021). With the extension of LSL treatment, the membrane permeability of both *S. aureus* and *P. aeruginosa* increased, resulting in a decrease in the intracellular ATP content, an increase in extracellular macromolecular substances, and final death of the cells. After LSL treatment for 4 h, the intracellular ATP loss rate of *S. aureus* was 92.92%, which was 6.58 times higher than that of untreated cells. For *P. aeruginosa*, the intracellular ATP loss rate was 52.60%, only 2.52 times higher than that of untreated cells. These results further suggest that the damaging effect of LSL on the cell membrane of *S. aureus* was much higher than that of *P. aeruginosa*.

## Conclusion

The LSL showed different inhibitory effects on different pathogenic bacteria. The best inhibitory effect performed by LSL was on *S. aureus*, followed by *P. aeruginosa*, and had no inhibitory effect against *E. coli* and *Lactobacillus* sp. To better understand the inhibition effect among LSL and different bacterial cell structures, the mechanism of LSL against *S. aureus* and *P. aeruginosa* was investigated. The results suggest that LSL had a greater influence on the cell membrane of *S. aureus*, while exhibited a stronger impact on the cell wall of *P. aeruginosa*. The obtained results endorsed the conclusion that LSL might be used for targeted treatment of special pathogenic bacteria and opportunistic pathogens.

## Data availability statement

The raw data supporting the conclusions of this article will be made available by the authors, without undue reservation.

## Author contributions

XM and DZ conceptualized the study. XM, TW, JS, and HZhu designed the experiments. DZ, XM, and HW supervised the study. HZ, TW, JS, and MJ performed the experiments. TW and HZ collected the data. TW carried out data analysis and statistics. JS, TW, HZ, and MJ contributed to materials and analysis tools. XM, TW, and HZ wrote the original draft. XM and TW wrote, revised, and edited the manuscript. All authors have read and approved the final manuscript.
